# Supplementary material for: Causal Relationship between Aging and Anorexia Nervosa: A White-Matter-Microstructure-Mediated Mendelian Randomization Analysis
Source: Biomedicines. 2024 Aug 16;12(8):1874. doi: 10.3390/biomedicines12081874 (PMC11351342; doi:10.3390/biomedicines12081874)
Supplement: Supplementary file 1 [file biomedicines-12-01874-s001.zip › Supplementary Figure/Figure S4/Singe SNP plot of MR analysis for TL to ubm-b-2083.pdf]

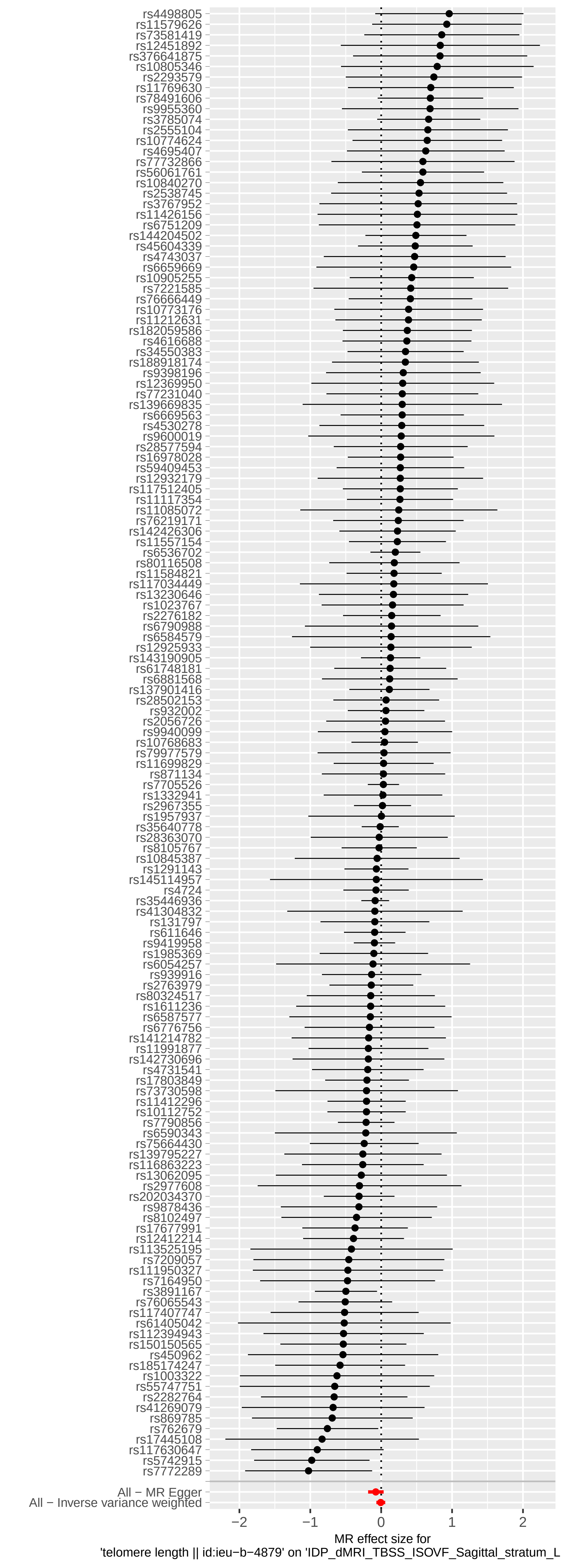

rs4498805
rs11579626
rs73581419
rs12451892
rs376641875
rs10805346
rs2293579
rs11769630
rs78491606
rs9955360
rs3785074
rs2555104
rs10774624
rs4695407
rs77732866
rs56061761
rs10840270
rs2538745
rs3767952
rs11426156
rs6751209
rs144204502
rs45604339
rs4743037
rs6659669
rs10905255
rs7221585
rs76666449
rs10773176
rs11212631
rs182059586
rs4616688
rs34550383
rs188918174
rs9398196
rs12369950
rs77231040
rs139669835
rs6669563
rs4530278
rs9600019
rs28577594
rs16978028
rs59409453
rs12932179
rs117512405
rs11117354
rs11085072
rs76219171
rs142426306
rs11557154
rs6536702
rs80116508
rs11584821
rs117034449
rs13230646
rs1023767
rs2276182
rs6790988
rs6584579
rs12925933
rs143190905
rs61748181
rs6881568
rs137901416
rs28502153
rs932002
rs2056726
rs9940099
rs10768683
rs79977579
rs11699829
rs871134
rs7705526
rs1332941
rs2967355
rs1957937
rs35640778
rs28363070
rs8105767
rs10845387
rs1291143
rs145114957
rs4724
rs35446936
rs41304832
rs131797
rs611646
rs9419958
rs1985369
rs6054257
rs939916
rs2763979
rs80324517
rs1611236
rs6587577
rs6776756
rs141214782
rs11991877
rs142730696
rs4731541
rs17803849
rs73730598
rs11412296
rs10112752
rs7790856
rs6590343
rs75664430
rs139795227
rs116863223
rs13062095
rs2977608
rs202034370
rs9878436
rs8102497
rs17677991
rs12412214
rs113525195
rs7209057
rs111950327
rs7164950
rs3891167
rs76065543
rs117407747
rs61405042
rs112394943
rs150150565
rs450962
rs185174247
rs1003322
rs55747751
rs2282764
rs41269079
rs869785
rs762679
rs17445108
rs117630647
rs5742915
rs7772289
All − MR Egger
All − Inverse variance weighted
−2
−1
0
1
2
MR effect size for
'telomere length || id:ieu−b−4879' on 'IDP_dMRI_TBSS_ISOVF_Sagittal_stratum_L
